# Relapsing Blastic Plasmacytoid Dendritic Cell Neoplasm With Neurologic Involvement

**DOI:** 10.7759/cureus.99491

**Published:** 2025-12-17

**Authors:** Zehra Rahman, Austin Haley, Shirley Gandhi, Abdullah Mohamed, Kevin Parza, JR Quan

**Affiliations:** 1 Internal Medicine, University of Florida College of Medicine – Jacksonville, Jacksonville, USA; 2 Internal medicine, University of Florida College of Medicine – Jacksonville, Jacksonville, USA; 3 Pathology and Laboratory Medicine, University of Florida College of Medicine – Jacksonville, Jacksonville, USA; 4 Oncology, University of Florida College of Medicine – Jacksonville, Jacksonville, USA

**Keywords:** dendritic cell neoplasm, hematologic malignancy, neurologic involvement of cancer, ocular manifestations of cancer, violaceous skin lesions

## Abstract

Blastic plasmacytoid dendritic cell neoplasm (BPDCN) is a rare and aggressive hematologic malignancy characterized by cutaneous, bone marrow, and central nervous system (CNS) involvement. Despite advances in therapy, prognosis remains poor, particularly in relapsed or refractory disease. We present the case of a woman in her 30s with a history of BPDCN diagnosed three months prior at an outside hospital following symptoms of blurry vision, headaches, night sweats, and weight loss. She achieved initial remission after AML-type induction chemotherapy (7+3 with cytarabine and idarubicin) but was lost to follow-up and received no consolidation therapy. She re-presented with worsening B symptoms, multiple new violaceous skin lesions, diffuse bone pain, ocular involvement, and progressive neurologic deficits. Diagnostic evaluation confirmed widespread relapse with infiltration of skin, lymph nodes, bone marrow, orbit, CNS, and lungs. She was treated with intrathecal chemotherapy and systemic daunorubicin/vincristine but demonstrated refractory disease and developed diffuse alveolar hemorrhage with cytologic confirmation of pulmonary BPDCN involvement. Given the aggressive relapse and complications, she is being transitioned to targeted therapy with tagraxofusp, with plans for eventual allogeneic stem cell transplantation pending clinical stability. This case highlights the fulminant course of relapsed BPDCN in a young patient, the limitations of conventional chemotherapy, and the urgent need for early consideration of novel targeted therapies. Recognition of atypical manifestations, including ocular and pulmonary involvement, is essential to guide timely diagnosis and management.

## Introduction

Dendritic cell neoplasms are a rare group of hematologic malignancies derived from precursor antigen-presenting cells, with subtypes including plasmacytoid, interdigitating, and follicular. Plasmacytoid dendritic cells (pDCs) normally serve as a critical component of the innate immune system, mounting rapid antiviral responses through type I interferon secretion. Blastic plasmacytoid dendritic cell neoplasm (BPDCN) is a rare and highly aggressive malignancy with an age-adjusted incidence of approximately 0.04 per 100,000 person-years [1]. It most commonly affects older males, with a median age at diagnosis of 60-70 years [2]. BPDCN typically involves the skin, presenting as violaceous tumors or plaques, but can also infiltrate the bone marrow, lymph nodes, spleen, and central nervous system (CNS). Occult CNS disease may be present at diagnosis even in the absence of neurologic symptoms, and relapse with CNS involvement is associated with a particularly poor prognosis [3]. Diagnosis relies on histopathology and immunophenotyping, with tumor cells expressing CD123, CD4, and CD56, while lacking lineage-defining myeloid or lymphoid antigens [4]. Relapse in BPDCN is often severe and treatment-refractory, particularly when neurologic involvement occurs [3]. We present a patient with severe, relapsing BPDCN complicated by neurologic involvement. This case underscores the importance of maintaining a broad differential diagnosis when evaluating atypical hematologic malignancies and highlights the need for individualized therapeutic strategies in rare disease presentations.

## Case presentation

This is a female patient in her 30s who was initially diagnosed with blastic plasmacytoid dendritic cell neoplasm (BPDCN) three months prior at an outside hospital. At that time, she was experiencing blurry vision, headaches, night sweats, and unintentional weight loss. Diagnosis was confirmed with bone marrow biopsy, and she underwent induction chemotherapy with 7+3 (cytarabine and idarubicin), reportedly achieving remission based on bone marrow flow cytometry. She was subsequently lost to follow-up and did not receive further consolidation or maintenance therapy. 

On presentation during this hospitalization, the patient reported that one month prior, she began developing new, painful violaceous lesions on her bilateral lower extremities, back, chest, and scalp, associated with severe diffuse pain. Two weeks prior to admission, she noted worsening night sweats and unintentional weight loss. She subsequently developed neurologic symptoms, including peripheral neuropathy of the hands and feet, as well as proximal muscle weakness. She also reported ocular symptoms of blurry vision, periorbital swelling, and left-sided ocular injection with green purulent discharge.

**Figure 1:**
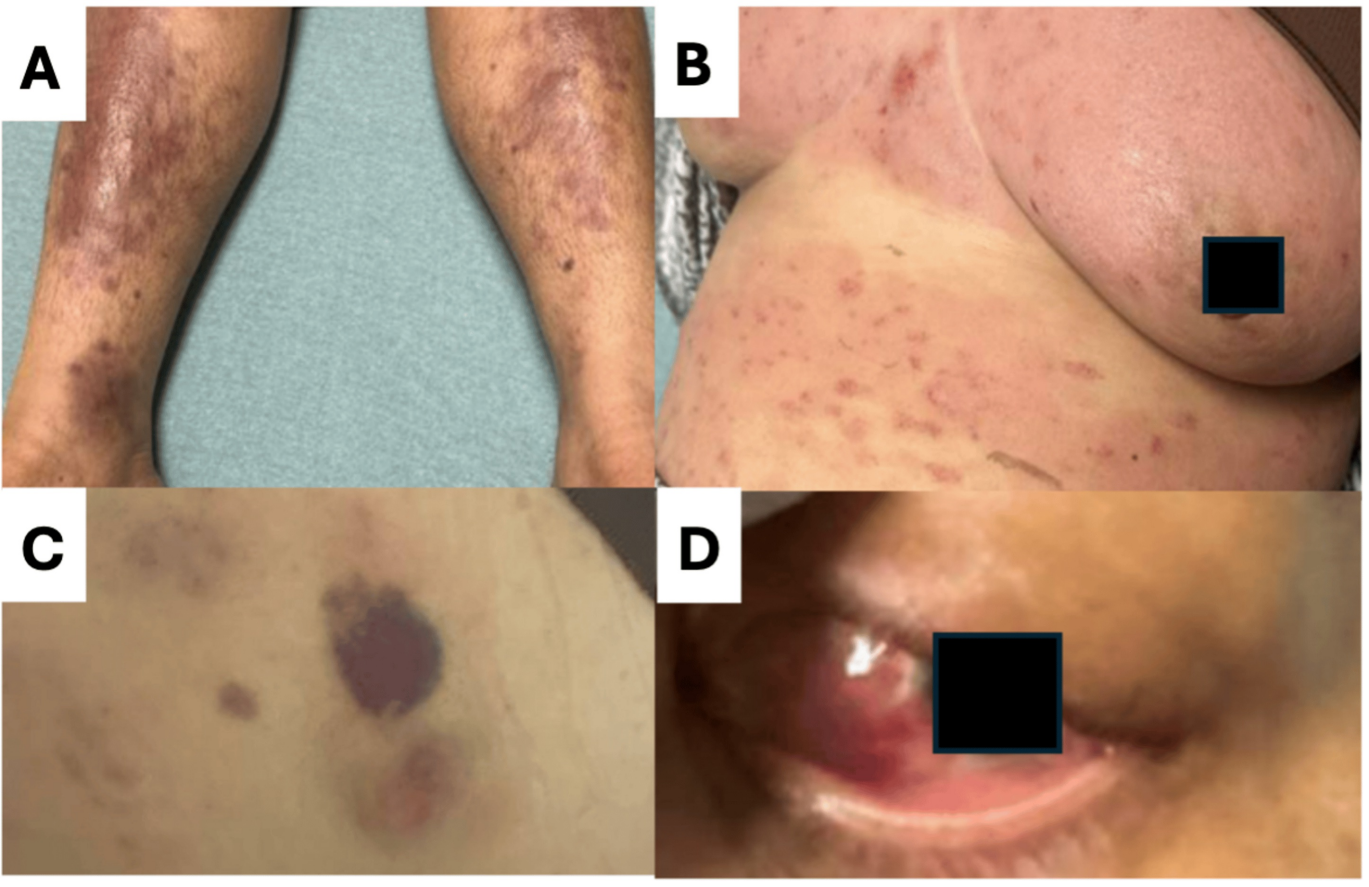
Physical examination findings. A) Bilateral violaceous skin lesions on the lower extremities. B) Skin lesions on the chest. C) Larger violaceous lesion on the left back. D) Left eye injection and chemosis.

Physical examination demonstrated intact extraocular movements; decreased muscle strength in both upper and lower extremities; intact reflexes; and decreased distal sensation in the upper and lower extremities. Dermatologic findings included large violaceous plaque-like lesions on the bilateral lower extremities and scattered purpuric lesions across the chest and back. The left eye showed marked injection and chemosis. Pertinent examination findings are shown in Figure 1.

**Figure 2:**
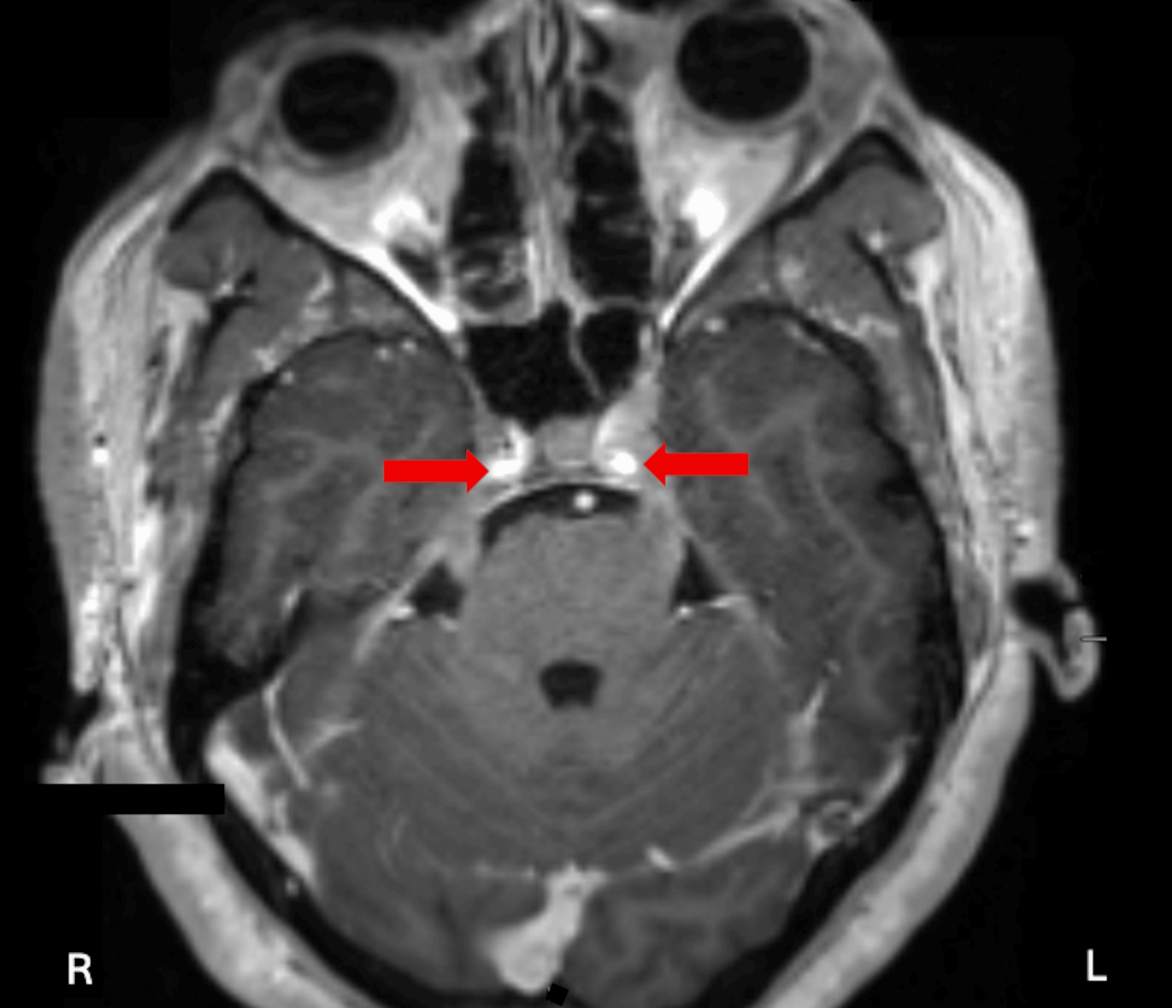
Axial post-contrast T1-weighted magnetic resonance imaging (MRI) of the brain demonstrates abnormal leptomeningeal enhancement along multiple cisternal cranial nerves with extension into the bilateral Meckel’s caves (red arrows).

Laboratory evaluation revealed leukocytosis (20,000/µL) with an absolute lymphocyte count of 13,000/µL, mild anemia with hemoglobin of 10.7 g/dL and thrombocytopenia of 32 × 10³/µL (Table 1). Leukocytosis with a lymphocyte-predominant differential is not typical in early BPDCN and more commonly reflects significant peripheral blood involvement in advanced or relapsed disease. Cytopenias, including anemia and thrombocytopenia, may be seen in BPDCN due to marrow involvement. She also had an elevated lactate dehydrogenase level at 1306 IU/L (Table 1), which is commonly found in aggressive hematologic malignancies. Magnetic resonance imaging (MRI) of the brain and orbits demonstrated abnormal leptomeningeal enhancement along multiple cisternal cranial nerves with extension into the bilateral Meckel’s caves, shown in Figure 2, concerning leptomeningeal spread. Given her lapse in therapy and current findings, there was a high suspicion of relapsed disease.

**Table 1:** Vital signs on admission and pertinent laboratory analysis.

Vitals Parameter	Result	Reference Range
Blood pressure	129/91 mmHg	Less than 120/80 mmHg
Temperature (oral)	36.8 C (98.3 F)	97°F (36.1°C) to 99°F (37.2°C)
Respiratory rate	18 bpm	12 to 20 breaths per minute
Oxygen saturation (Spo2)	97%	95% to 100%
Laboratory Parameter	Result	Reference Range
White Blood Cell Count (WBC)	20,000 cells/µL	4,000 - 11,000 cells per microliter
Absolute Neutrophil Count (ANC)	4,000 cells/µL	1,500 and 8,000 cells per microliter
Absolute Lymphocyte Count (ALC)	13,000 cells/µL	1,000 - 4,800 cells per microliter
Hemoglobin (Hb)	10.7 g/dL	12.0 - 16.0 grams per deciliter (female) 13.5 - 17.5 grams per deciliter (male)
Platelet count	32 × 10³/µL	140 - 440 × 10³/µL
Lactate dehydrogenase (LDH)	1306 IU/L	126 - 266 IU/L

**Figure 3:**
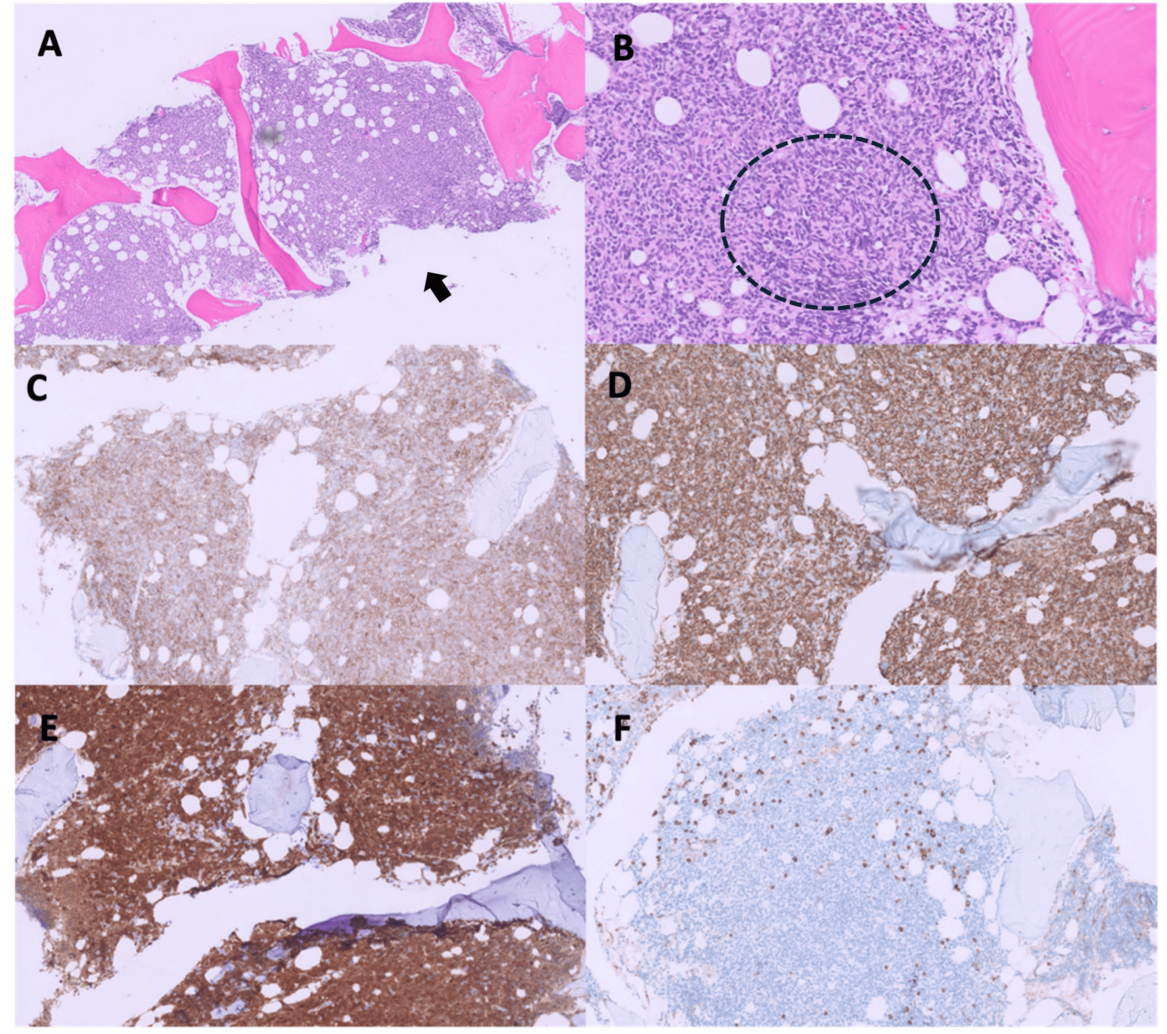
Histopathologic and immunohistochemical findings from bone marrow biopsy. (A) H&E-stained section (×5) shows near-total replacement of normal hematopoietic elements by a monotonous infiltrate (arrow). (B) Higher magnification (×10) highlights small- to intermediate-sized tumor cells with scant cytoplasm, fine blastic chromatin, and occasional spindled morphology (circle). (C–F) Immunohistochemistry shows diffuse expression of CD4 (×10, panel C), CD56 (×10, panel D), and TCL1 (×10, panel E) in neoplastic cells, with negative staining for myeloperoxidase (MPO) (×10, panel F).

**Figure 4:**
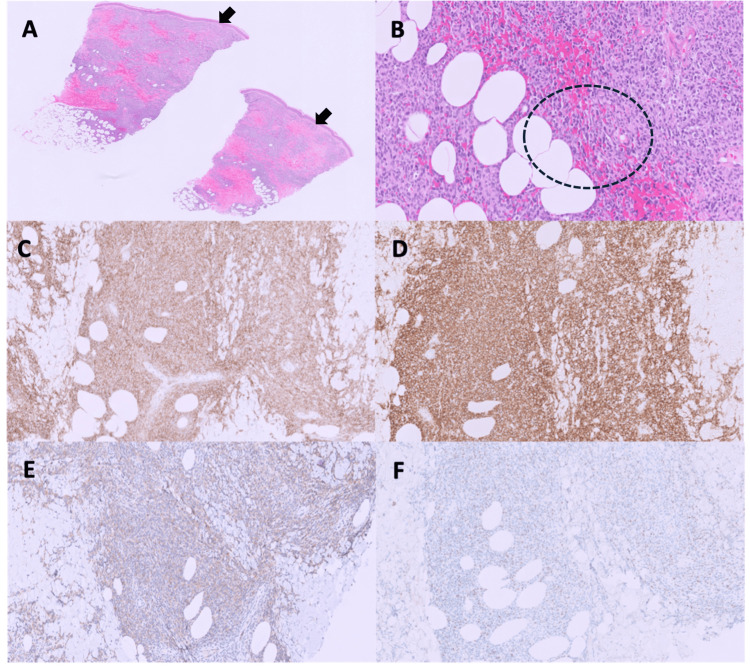
Histopathologic and immunohistochemical findings from skin biopsy. (A) H&E-stained section (×2) of the wedge skin biopsy shows diffuse dermal infiltration by small blue tumor cells arranged in a sheet-like growth pattern, sparing the epidermis and separated from it by a narrow grenz zone (arrows). (B) Higher magnification (×10) demonstrates subcutaneous infiltration by atypical blastic ells (dashed circle). The tumor cells display dense, homogeneous, blast-like chromatin. (C–F) The cutaneous infiltrate shows strong immunoreactivity for CD4 (×10, panel C) and CD56 (×10, panel D), weak expression of CD123 (×10, panel E), and negativity for TdT (×10, panel F).

**Figure 5:**
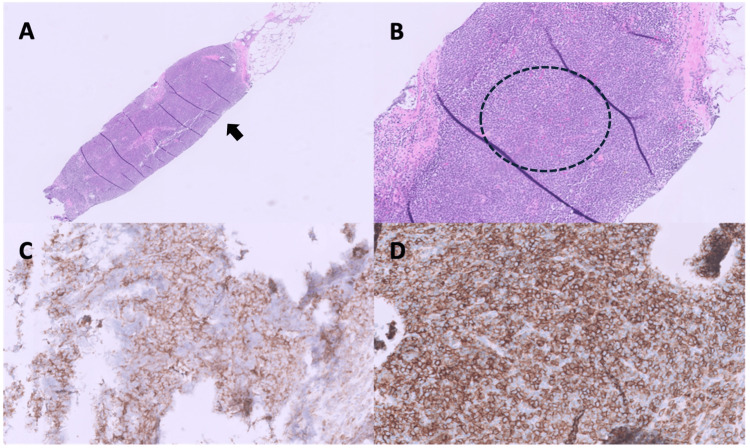
Histopathologic and immunohistochemical findings from lymph node biopsy. (A) H&E-stained section (×2) from an ultrasound-guided core biopsy of the right inguinal lymph node shows effacement of the nodal architecture by an atypical cellular infiltrate (arrow). (B) Higher magnification (×10) highlights infiltrates of atypical blastoid cells with dense chromatin and scant cytoplasm (dashed circle). (C, D) Immunohistochemistry confirms diffuse expression of CD4 (×20, panel C) and CD56 (×20, panel D) in the lymph node infiltrate.

Bone marrow biopsy confirmed persistent BPDCN with approximately 80-90% marrow involvement (Figure 3). Peripheral blood flow cytometry showed 76% BPDCN involvement. Skin biopsy from a right lower extremity lesion demonstrated extensive dermal and subcutaneous infiltration by atypical medium to large cells with irregular nuclear contours and spindled morphology, consistent with BPDCN (Figure 4). Flow cytometry from a right inguinal lymph node biopsy showed 57% involvement by atypical CD4(+)/CD56(+) cells (Figure 5). Cerebral spinal fluid (CSF) flow cytometry confirmed CNS infiltration with 84% BPDCN involvement. Overall, findings indicated severe, relapsed BPDCN with infiltration of the CSF, orbit, bone marrow, lymph nodes, and skin.

The patient underwent five weekly intrathecal chemotherapy treatments alternating methotrexate and cytarabine until CSF flow cytometry showed clearance of disease. Although tagraxofusp is increasingly used as frontline therapy for BPDCN, daunorubicin and vincristine were selected at relapse because the patient required immediate treatment and access to tagraxofusp was limited by insurance-related barriers. She also began cycle 1 of daunorubicin and vincristine, with doses reduced by 50% after day 1 due to transaminitis and hyperbilirubinemia. On cycle 1, day 22, both agents were held for persistent cytopenias and hepatotoxicity. Aspartate aminotransferase (AST) peaked at 38 IU/L, and alanine aminotransferase (ALT) peaked at 557 IU/L. Repeat bone marrow biopsy two months after treatment again demonstrated persistent BPDCN with 80-90% involvement, consistent with refractory disease.

Her hospital course was further complicated by diffuse alveolar hemorrhage (DAH), evidenced by diffuse alveolar infiltrates on chest imaging and confirmed by bronchoalveolar lavage (BAL) with bloody aliquots. At that time, coagulation studies showed only mild abnormalities, with an INR of 1.3 and PT of 14.8 seconds, while fibrinogen was elevated at 680 mg/dL, indicating an acute-phase response rather than a consumptive coagulopathy. BAL cytology revealed BPDCN involvement, with positivity for CD45, CD56, E-cadherin, CD123, and TCL1, confirming pulmonary infiltration. Given her multi-organ involvement and poor marrow response, plans were made to initiate targeted therapy with tagraxofusp (a CD123-directed therapy) and pursue allogeneic bone marrow transplantation. However, initiation of intensive therapy was deferred pending clinical stabilization and improvement of DAH.

## Discussion

BPDCN carries a poor prognosis, particularly in the relapsed or refractory setting. Median overall survival is estimated at 12-24 months, with worse outcomes observed in patients of older age, those with bone marrow involvement, and those harboring certain genetic abnormalities [5]. Although BPDCN typically presents in males older than age 60 years, our patient represents an unusual case of an aggressive relapse in a woman in her 30s, emphasizing that younger patients are not spared from severe disease trajectories [6].

Historically, treatment approaches for BPDCN have been modeled after acute leukemia regimens, often incorporating intrathecal therapy into the regimen alongside systemic chemotherapy for patients with neuromeningeal disease. However, these strategies have achieved limited long-term success with high rates of relapse [5]. Our patient initially received AML-based induction chemotherapy (7+3) and achieved reported remission, but she was lost to follow-up and did not receive consolidative therapy. On re-presentation only three months later, she had extensive multi-organ relapse involving the skin, bone marrow, lymph nodes, orbit, and CNS. CNS involvement is increasingly recognized as both occult at diagnosis and a common site of relapse, and it is associated with poor prognosis [3]. The presence of neurologic manifestations, including peripheral neuropathy, ocular involvement, and confirmed leptomeningeal disease in our patient, exemplifies this aggressive pattern [7].

Management of relapsed BPDCN remains particularly challenging. Our patient underwent intrathecal chemotherapy with methotrexate and cytarabine, achieving temporary clearance of CSF involvement, along with systemic therapy. Daunorubicin and vincristine were initially selected due to the need for rapid cytoreduction at relapse and because access to tagraxofusp was restricted by insurance-related barriers. However, she rapidly demonstrated refractory disease with persistent high-level marrow infiltration, complicated further by pulmonary involvement with diffuse alveolar hemorrhage (DAH) confirmed to be infiltrated by BPDCN. These findings highlight both the aggressive biology of BPDCN and the limitations of conventional chemotherapy in achieving durable remission.

Allogeneic stem cell transplantation in first remission remains a potentially curative approach, but eligibility is often restricted by patient comorbidities and performance status [8]. In our patient, transplant is being considered once clinical stability is achieved, but her course illustrates how complications such as treatment-related toxicities and DAH can delay or prevent timely progression to curative strategies. More recently, tagraxofusp, a CD123-directed cytotoxin, has emerged as the first approved targeted agent for BPDCN and is now considered first-line therapy in eligible patients [9]. Plans are underway to transition our patient to tagraxofusp therapy, reflecting the evolving landscape of BPDCN management. Other novel approaches under investigation include CAR-T therapy, hypomethylating agents, and additional CD123-directed therapies, though these remain experimental [10].

This case underscores the therapeutic challenges in BPDCN, particularly when relapse is complicated by widespread systemic and CNS involvement. It highlights the importance of early recognition of neurologic manifestations, the limitations of traditional leukemia-based chemotherapy regimens, and the urgent need for novel targeted therapies and individualized treatment strategies in high-risk disease presentations.

## Conclusions

BPDCN remains a rare and aggressive hematologic malignancy with limited therapeutic options and a high risk of relapse, particularly when the central nervous system is involved. Our case underscores the importance of considering BPDCN in the differential diagnosis of atypical hematologic or neurologic presentations and highlights the challenges clinicians face in balancing disease control with treatment-related risks. As novel targeted and cellular therapies continue to emerge, timely recognition, risk stratification, and individualized treatment planning will be essential to improving outcomes in this devastating disease.
